# Evaluation of Safety in Horizontal Curves of Roads Using a Multi-Body Dynamic Simulation Process

**DOI:** 10.3390/ijerph17165975

**Published:** 2020-08-17

**Authors:** Amir Saman Abdollahzadeh Nasiri, Omid Rahmani, Ali Abdi Kordani, Nader Karballaeezadeh, Amir Mosavi

**Affiliations:** 1 Department of Civil Engineering, South Tehran Branch, Azad University, Tehran 1584715414, Iran; st_as_nasiri@azad.ac.ir; 2 Faculty of Civil Engineering, Shahrood University of Technology, Shahrood 3619995161, Iran; Omid.rahmani@shahroodut.ac.ir (O.R.); n.karballaeezadeh@shahroodut.ac.ir (N.K.); 3 Department of Civil Engineering, Imam Khomeini International University, Qazvin 3414896818, Iran; aliabdi@eng.ikiu.ac.ir; 4 Faculty of Civil Engineering, Technische Universität Dresden, 01069 Dresden, Germany; 5 Institute of Research and Development, Duy Tan University, Da Nang 550000, Vietnam; 6 Department of Informatics, J. Selye University, 94501 Komarno, Slovakia

**Keywords:** road safety, transport safety, accident analysis and prevention, public health risks, horizontal curves, safety margin parameter, road traffic injuries, world health organization, multi-body dynamic simulation, mobility, transport, traffic safety, road design

## Abstract

Road transportation poses one of the significant public health risks. Several contributors and factors strongly link public health and road safety. The design and advancement of higher-quality roads can significantly contribute to safer roads and save lives. In this article, the safety aspect of the roads’ horizontal curves under the standard of the American Association of State Highway Transportation Officials (AASHTO) is evaluated. Several factors, including vehicle weight, vehicle dimensions, longitudinal grades, and vehicle speed in the geometric design of the horizontal curves, are investigated through a multi-body dynamic simulation process. According to the AASHTO, a combination of simple circular and clothoid transition curves with various longitudinal upgrades and downgrades was designed. Three vehicles were used in this simulation, including a sedan, a bus, and a 3-axle truck. The analysis was based on the lateral friction between the tire and the pavement and also the safety margin parameter. The results showed that designers must differentiate between light and heavy vehicles, especially in curves with a high radius. Evaluation of longitudinal grade impacts indicated that the safety margin decreases when the vehicle is entering the curve. Safety margin reduction on the clothoid curve takes place with a lower grade toward the simple circular curve. By increasing the speed, the difference between lateral friction demand obtained from simulation and lateral friction demand proposed by AASHTO grows. The proposed novel methodology can be used for evaluating road safety.

## 1. Introduction

Several studies indicate that most road accidents occur at horizontal curves [1,2,3,4,5,6]. At horizontal curves, the risk of accidents is significantly higher than that in other sections of the road [7]. Several critical factors are often involved in road accidents, i.e., road characteristics, vehicle performance, driver behavior, and environmental factors [8]. Among these factors, the geometric characteristic is investigated in this study. Improvement of the road’s geometric design and proper evaluation of its dependent variables would significantly contribute to designing safer roads for reducing the number of accidents [9]. The curve radius is the most critical parameter in the geometric design of horizontal curves [10]. Recent studies indicate that the number of accidents has a direct connection with the curve radius [8,10,11]. Based on “American Association of State Highway Transportation Officials (AASHTO) Green Book 2018” [12], the minimum radius of a horizontal curve can be calculated by as follows.

(1) $$R=\frac{V^{2}}{127\left(e\;+\;f\right)}$$

where R is curve radius (m), V is speed (km/h), e is superelevation, and f is lateral friction. This equation is known as the point-mass model. Although Equation (1) is the basis of the geometric design in horizontal curves, the authors believe that this equation does not consider several important aspects. For example, this model does not regard the changes in the weight and dimensions of vehicles. The authors believe that the values of friction in tires can be different for various vehicles [13,14]. On the other hand, AASHTO only considered cross-sectional slopes (superelevation) in this model, whilst, longitudinal grades sometimes coincide with horizontal curves. In such states, the model can not apply the impact of longitudinal grades on the radius. Moreover, the point-mass model determines the radius for a specific speed (V). However, the behavior of the driver does not follow a constant pattern, and sometimes the speed of the vehicle is more or less than the designed value. Unfortunately, this difference in the driver’s behavior was not considered in the model. Regarding the above, it is evident that the main concern of authors is safety because a perfect, flawless geometric design can increase safety. Therefore, this paper has focused on assessing the above vague points to improve safety in the horizontal curves.

Recently, many studies have been carried out to measure the safety of horizontal and vertical curves. Instead of applying the lateral friction as the only parameter related to the pavement in designing curves, a more practical parameter called the safety margin has been used [15]. The safety margin is defined as the difference between friction supply and friction demand. Safety margin analysis is a suitable method to evaluate the safety of a curve in terms of geometric performance and pavement friction [16]. Due to changes in vehicle speed and superelevation along the curve, the safety margin should be evaluated throughout the curve [17]. The safety margin obtained from the tire–pavement lateral friction has been introduced as an efficient method for the proper assessment of the design theory and geometric characteristics of the horizontal curve. Some studies have estimated the safety of horizontal curves based on tire–pavement friction with the help of the ADAMS/Car (Automated Dynamic Analysis of Mechanical System) simulator and the safety margin theory [18]. Recent studies about the estimation of the safety margin have mainly focused on friction supply and friction demand on horizontal curves [6,10,15,16,19,20,21]. On the other hand, the use of the clothoid transition curve between the tangent and circular curve has been suggested to increase the safety margin against skidding, especially when superelevation exceeds 12% [16]. Transition curves improve the safety of horizontal curves because they help to gradually implement superelevation and centrifugal force. Therefore, the application of clothoid transition curves is another way to improve the safety of horizontal curves.

In this paper, the authors simulated two commonplace types of horizontal curves by using ADAMS/Car: simple circular and clothoid–circle–clothoid. They aimed to examine the safety of these types of curves based on the safety margin parameter and lateral friction.

The first innovation of this paper is the accurate assessment of the proposed lateral friction by AASHTO. For all types of vehicles, longitudinal grades, and driver behaviors (various speeds), AASHTO recommends constant friction. However, this paper examines the changes in weight and dimensions of vehicles and the impacts of these variations on the friction value. Furthermore, this study assesses the effect of longitudinal grade and changes in speed. The other novelty of this study is to present a novel methodology for evaluating safety in horizontal curves. This methodology is named the multi-body dynamic simulation process. In this methodology, the safety of the transition curve can be assessed by using the dynamic response of vehicles. The methodology uses the safety margin parameter, based on the side friction, to examine safety. By using this methodology, engineers can evaluate every type of horizontal curve. Therefore, the methodology makes easy the evaluation of safety in horizontal curves for engineers.

## 2. Methods and Materials

### 2.1. The Multi-Body Dynamic Simulation Process

**Figure 1:**
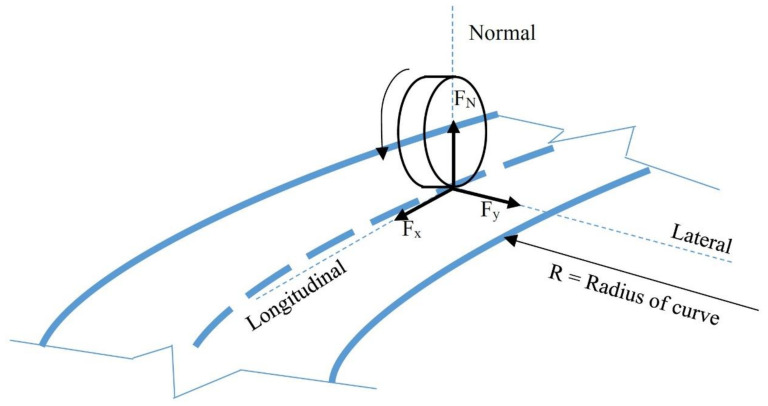
Definition of forces in tire-pavement interaction.

**Figure 2:**
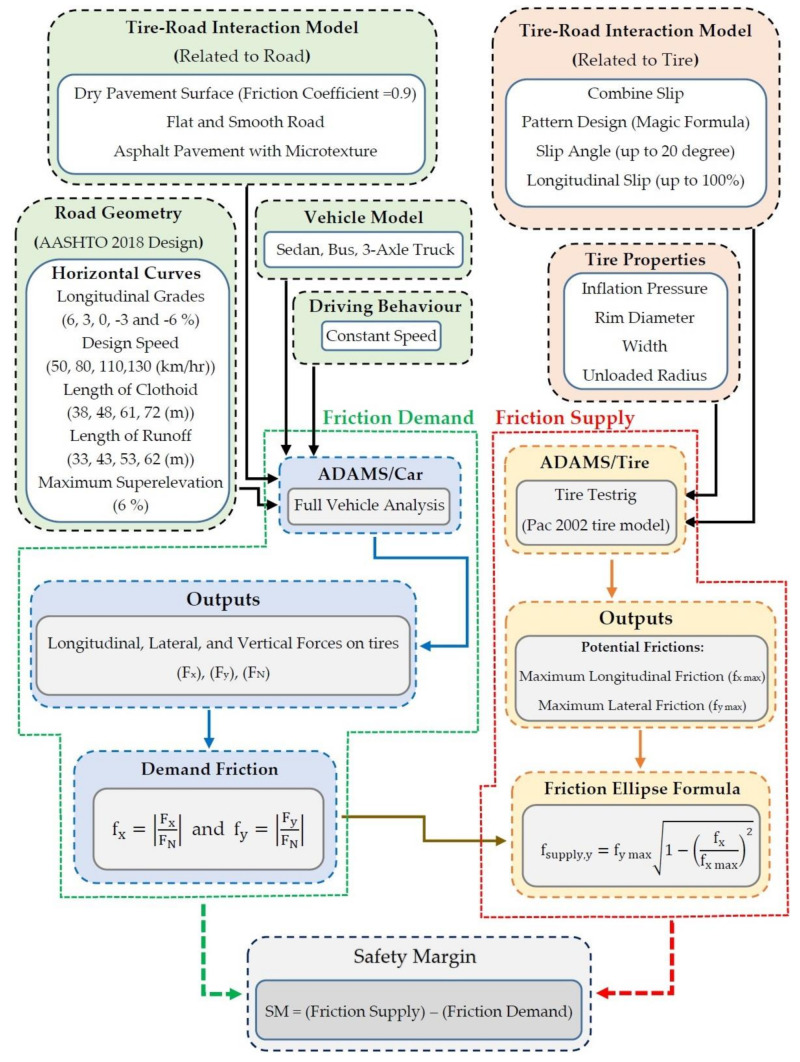
Research framework using the multi-body dynamic simulation.

In this study, a multi-body dynamic simulation process has been applied. The objective of this simulation is to investigate the safety margin for the geometric design of horizontal curves on roads. The safety margin is the difference of the available (supply) and demand tire–pavement friction for vehicles that track a horizontal curve [16]. The exact safety margin for the designed road is obtained from the lateral and longitudinal friction factors between the tire and the pavement. Figure 1 shows the form of the forces in the tire–pavement interaction. Additionally, Figure 2 presents the research framework using a multi-body dynamic simulation. In Figure 1; Figure 2, F_x_, F_y_, F_N_, f_xmax_, f_ymax_, f_ydemand_, and f_ysupply_ are longitudinal force, lateral force, normal force, the peak longitudinal friction, the peak lateral friction, lateral friction factor, and friction available between the pavement and tires for preventing skidding, respectively.

### 2.2. Modeling

The open-loop process consists of two essential components: full-vehicle modeling and road modeling. The full-vehicle modeling in ADAMS/Car consists of several components such as car body, vehicle suspension, braking, and steering (driver behavior in event builder of ADAMS/Car), etc. ADAMS is a simulation software for predicting the mechanical system performance, collision detection, motion range, and peak load. The quality of the simulation in ADAMS is high, and the modeling is complex [6]. The road was modeled by classical equations for circle and clothoid (also known as spiral) curves.

#### 2.2.1. Circular Curve Modeling in ADAMS/Car

Multi-quadric interpolation functions were used for creating the three-dimensional circular horizontal curve in the ADAMS/Car simulator. The mathematical forms of these functions are presented in Equations (2) and (3). In Equation (2), there is a one-variable multi-quadric interpolation function that is applied to handle pavement smoothness. Equation (3) shows a bivariate interpolation function, which is applied to handle 3D road smoothness and elevation [22,23].

(2) $$F\left(x\right)=\sum_{j=1}^{n}a_{j}{\left[{\left(x-x_{j}\right)}^{2}+R^{2}\right]}^{0.5},$$

(3) $$F\left(x,y\right)=\sum_{j=1}^{n}a_{j}{\left[{\left(x-x_{j}\right)}^{2}+{\left(y-y_{j}\right)}^{2}+R^{2}\right]}^{0.5},$$

where F(x) is one-variable multi-quadric interpolation function, a_j_ is an undetermined coefficient, x_j_ is the jth centerline coordinate scattered on the horizontal curve, R is the smooth factor, x is interpolation point between x_j_ and x_j + 1_, and F(x,y) is bivariate interpolation function.

#### 2.2.2. Clothoid Curve Modeling in ADAMS/Car

There are two methods for designing the clothoid curve in ADAMS/Car:

- The first method, introduced by Abramowitz and Stegun [24],
- The second method, introduced by Vazquez-Mendez and Casal [25].

Although the second method is more precise, the first method (also known as the classic method) can be accepted when the ratio of “(spiral length)/(2 × circular curve radius)” is less than 3. The designed values for simulated curves in this study are presented in Table 1. Based on this table, the classical method has been used for modeling the clothoid.

For each clothoid curve, parameter A can be calculated by basic transformations from the standard clothoid (see Equations (4)–(7)). In solving integrals in Equations (4) and (5), the following 3-step algorithm is widely used [25]:

1. Consider the McLaurin polynomials of a certain degree for sin(x) and cos(x).
2. Replace the values of the integrated solution in Equations (6) and (7) in McLaurin polynomials at the point $\tau^{2}/\left(2A^{2}\right)$.
3. Use Barrow’s rule to compute the result of integral.

The polynomials of degree 11 for sin(x) and degree 12 for cos(x) are calculated as an example, the following approximations are estimated (Equations (6) and (7)) [24]:

(4) $$\hat{\;x\;}\left(s\right)=\int_{0}^{s}\sin\left(\frac{\tau^{2}}{2A^{2}}\right)d\tau ,\;s\in \left[0,L\right]$$

(5) $$\hat{\;y\;}\left(s\right)=\int_{0}^{s}\cos\left(\frac{\tau^{2}}{2A^{2}}\right)d\tau ,\;s\in \left[0,L\right]$$

(6) $$\hat{\;x\;}\left(s\right)\approx \frac{s^{3}}{2!3A^{2}}-\frac{s^{7}}{3!7{\left(\sqrt{2}A\right)}^{6}}+\frac{s^{11}}{5!11{\left(\sqrt{2}A\right)}^{10}}-\frac{s^{15}}{7!15{\left(\sqrt{2}A\right)}^{14}}+\frac{s^{19}}{9!19{\left(\sqrt{2}A\right)}^{18}}-\frac{s^{23}}{11!23{\left(\sqrt{2}A\right)}^{22}},\;s\in \left[0,\;L\right]$$

(7) $$\hat{\;y\;}\left(s\right)\approx s-\frac{s^{5}}{2!5{\left(\sqrt{2}A\right)}^{4}}+\frac{s^{9}}{4!9{\left(\sqrt{2}A\right)}^{8}}-\frac{s^{13}}{6!13{\left(\sqrt{2}A\right)}^{12}}+\frac{s^{17}}{8!17{\left(\sqrt{2}A\right)}^{16}}-\frac{s^{21}}{10!21{\left(\sqrt{2}A\right)}^{20}},\;s\in \left[0,\;L\right]$$

where A is the parameter of clothoid, L is the maximum length of clothoid, S is the length of clothoid ($S\in \left[0,\;L\right]$), and τ is dummy variable.

#### 2.2.3. Designed Parameters in Horizontal Curves

**Figure 3:**
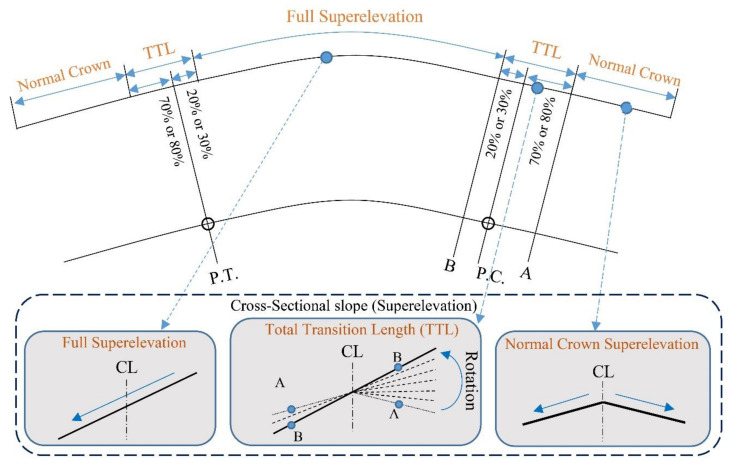
Implementation of superelevation in simple circular curves. PC–entrance of circle curve, PT–end of the circle curve, CL–center line of road cross-section

**Figure 4:**
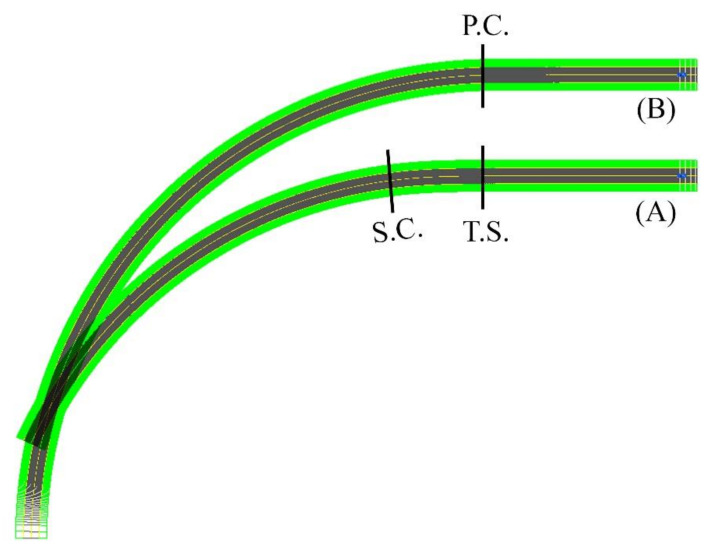
(**A**) Clothoid–circle-clothoid curve and (**B**) Simple circular curve. PC–entrance of the circle curve, TS–enterance of clothoid-circle curve, SC: entrance of circle part in clothoid-circle-clothoid curve.

Geometric design characteristics, including design speed, superelevation, and horizontal curve radius, under the AASHTO regulations, are listed in Table 2. Other geometric design characteristics, including longitudinal grade, lateral friction factor, the road width for both simple circular and clothoid–circle–clothoid curves are shown in Table 3. Based on AASHTO Green Book 2018, the authors designed four different scenarios with various design speeds, including 50, 80, 110, and 130 km/h. In all scenarios, the superelevation is 6%. Moreover, different longitudinal grades (−6%, −3%, 0%, +3%, and +6%) were combined with horizontal curves. In fact, the computational theory of safety margin and the dynamic response of vehicles were employed for evaluating the simple circular and clothoid–circle–clothoid curves. On circular curves, 80% of the superelevation is applied before the curve at a speed of 20 to 70 km/h. At a speed of 80 to 130 km/h, 70% of the superelevation is applied before the curve as schematically shown in Figure 3 [12]. Total transition length (TTL) is indicated in Figure 3. By using Figure 4, the schematic comparison of curves is possible.

### 2.3. Vehicle Modeling

**Figure 5:**
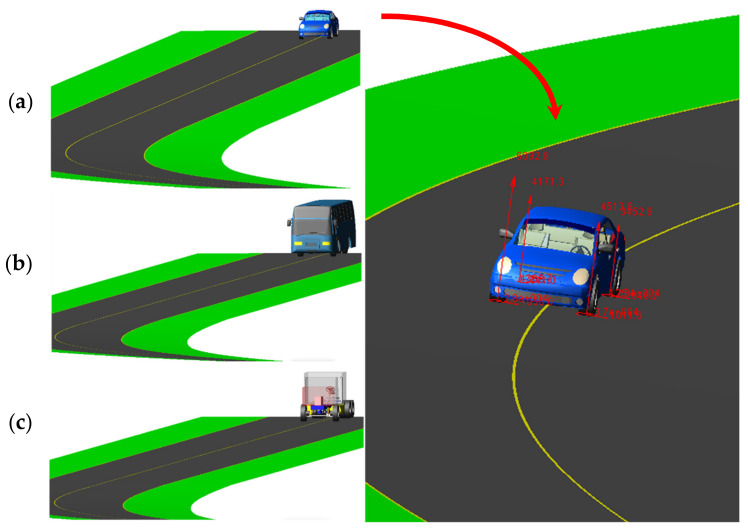
Designed vehicles used in the ADAMS/Car for this study: (**a**) sedan, (**b**) bus, and (**c**) 3-axle truck.

Three different vehicles, with different sizes and dynamic parameters, were used in the simulations of this study. These vehicles include a sedan Rear Wheel Drive (RWD), a rigid bus, and a 3-axle truck unit. Figure 5 depicts the vehicles used in simulations. These vehicles were picked from among the default vehicles of the ADAMS/Car simulation software. Table 4 presents the size, weight, and mass center of these three vehicles, which is an adaptation from [26].

### 2.4. The Tire–Road Friction Modeling

The road–tire friction is defined as the friction between the road surface and vehicle tires for preventing skidding on horizontal curves. The maximum lateral friction factor is used at the skidding threshold [16]. The friction force between the road and the tire is originated from the sideslip between the elastomer and the road surface [27].

According to the ellipse formula, f_xmax_ and f_ymax_ must be first estimated to calculate the friction supply (f_supply_). Many factors affect the maximum lateral force acting on the tire and the maximum lateral friction factor including the normal and longitudinal forces on the tire, road surface condition (dry, wet, snowy, icy, etc.), the vertical load acting on the tire, speed, tire condition (new, worn-out), and tire composition [16]. According to adoption from [26], all of the parameters are shown in Table 5. When tire properties are characterized, the tire models can predict tire friction by combining bending and braking. The elliptical curves represent the forces in the tire [16].

The Magic Formula is a mathematical formula for expressing the basic properties of the tire. There are two primary forms of the Magic Formula including sinus (sin) and cosine (cos) (see Equations (8) and (9)) [28].

(8) $$Y\left(x\right)=D\times \sin\left[C\times \arctan\left\{\left(B\times x\right)-E\left(\mathrm{Bx}-\arctan\left(B\times x\right)\right)\right\}\right],$$

(9) $$Y\left(x\right)=D\times \cos\left[C\times \arctan\left\{\left(B\times x\right)-E\left(\mathrm{Bx}-\arctan\left(B\times x\right)\right)\right\}\right],$$

where Y(x) may be the longitudinal force (F_x_), lateral force (F_y_), or aligning torque, and correspondingly x is the slip ratio κ (driving or braking conditions) or wheel slip angle α. Lateral wheel slip is defined as the ratio of the lateral and the forward movement of the wheel [28]. D is the peak factor (factor determines of the characteristic), C is the shape factor (factor determines the part used of the sine and, therefore, mainly influences the shape of the curve), B is the stiffness factor (factor stretches the curve), and E is the curvature factor (this factor can modify the characteristic around the peak of the curve).

For pure slip conditions, the lateral force (F_y_) and longitudinal force (F_x_) as a function of the lateral slip α and longitudinal slip κ, respectively, have a similar shape. Because of the sine arctangent combination, the basic Magic Formula equation is capable of describing this shape [28,29]:

(10) $$F_{x}={\;F}_{x0}\left(\kappa ,{\;F}_{z},\gamma \right)\to \mathrm{Pure}\;\mathrm{Slip},$$

where κ is longitudinal slip, $F_{z}$ is the normal wheel load, and γ is the inclination angle.

(11) $$F_{x}={\;F}_{x0}.G_{\mathrm{xa}}\left(\alpha ,\kappa ,{\;F}_{z}\right)\to \mathrm{Combined}\;\mathrm{slip},$$

where α is the slip angle for longitudinal force (F_x_). Therefore,

(12) $$F_{x}={\;D}_{x\alpha }\times \cos\left[C_{x\alpha }\times \arctan\left\{B_{x\alpha }\alpha_{s}-E_{x\alpha }\left(B_{x\alpha }\alpha_{s}-\arctan\left(B_{x\alpha }\alpha_{s}\right)\right)\right\}\right].$$

## 3. Result and Discussion

### 3.1. Maximum Lateral and Longitudinal Frictions

**Figure 6:**
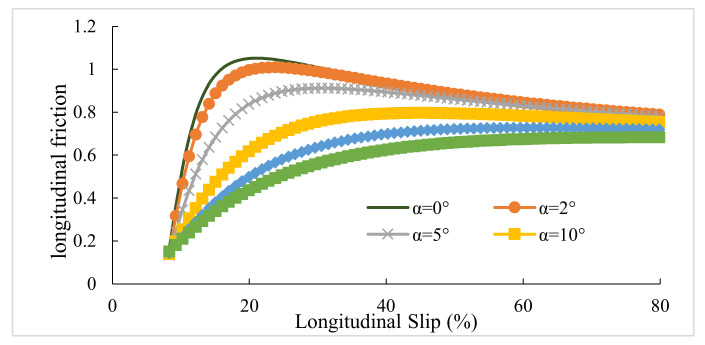
The maximum calculated longitudinal friction (f_x max_) in various lateral slip angles for the pac2002-235-40R18 tire.

**Figure 7:**
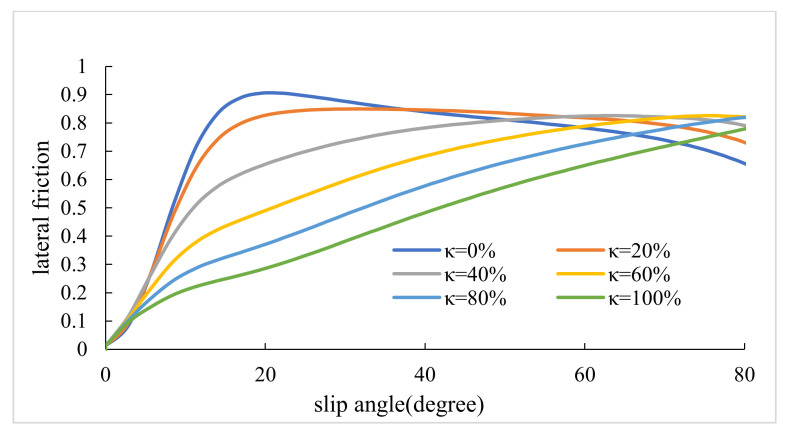
The maximum calculated lateral friction (f_y max_) in various slip ratios for the pac2002-235-40R18 tire.

**Figure 8:**
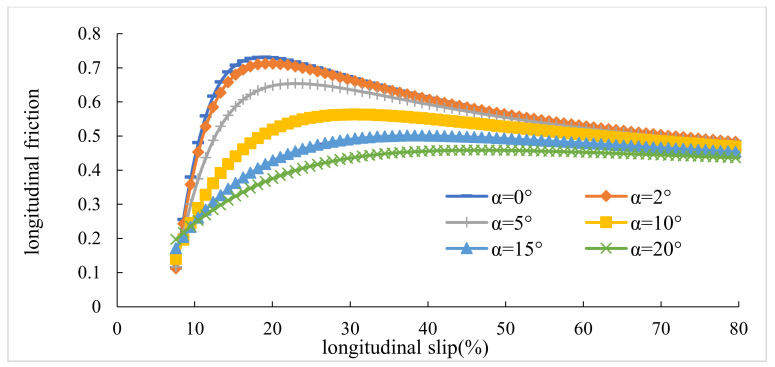
The maximum calculated longitudinal friction (f_x max_) in various lateral slip angles for the pac2002-255-40R19 tire.

**Figure 9:**
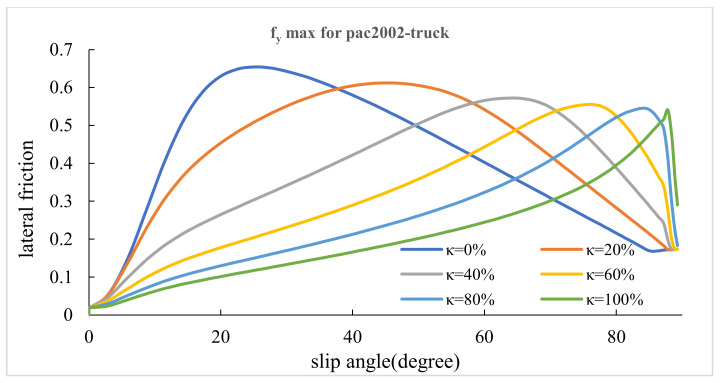
The maximum calculated lateral friction (f_y max_) in various slip ratios for the pac2002-255-40R19 tire.

This section presents the values of lateral and longitudinal frictions for various lateral slip angles (α) and slip ratios (κ). Firstly, slips were combined. Afterward, the diagrams of slip–friction and slip angle–friction were obtained for both lateral and longitudinal conditions. Figure 6, Figure 7, Figure 8, Figure 9 represent the results for tires pac2002-235-40R18 and pac2002-255-40R19. In these diagrams, peak points show the potential of longitudinal and lateral forces between the tire and road. After examining the outputs of the designed tires, namely pac2002-235-40R18 and pac2002-255-40R19 tires, f_xmax_ and f_ymax_ were obtained as shown in Table 6. The values obtained are inserted in the friction ellipse formula [30].

### 3.2. Lateral Friction Demand

**Figure 10:**
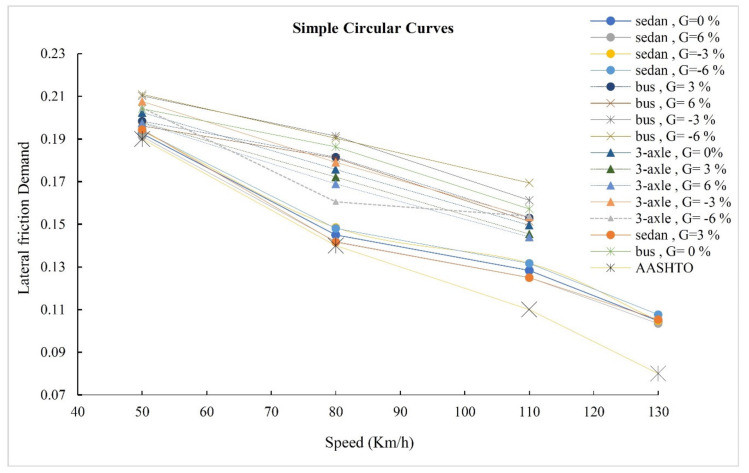
Lateral friction demand in simple circular curves.

This section compares the lateral friction demand obtained from simulation with the maximum friction factors proposed by AASHTO. Figure 10 displays variation in the lateral friction demand versus the speed for simple circular curves. In terms of Equation (1), the horizontal axis of Figure 9 involves the radius of the curve. Based on Equation (1), speed and radius have a direct correlation together. For all three vehicles, lateral friction demand is close to those proposed by AASHTO at low speeds. By increasing the speed and consequently increase the radius, the difference between simulation outputs and AASHTO values increase. Among all the vehicles, the lateral friction demand of sedan has the most similarity with the values proposed by AASHTO. Albeit, the difference between the sedan and AASHTO values is noticeable for high speeds. The lateral friction demand for the bus and the 3-axle truck is very close, but the friction values for the bus are a bit higher. As can be seen in Figure 10, the lateral friction demand proposed by AASHTO for heavy vehicles does not adjust with the results of the simulation. Therefore, Figure 9 verifies that the geometric design criteria of the road must differ according to the dynamic response of the vehicles to obtain a desirable safety margin for the vehicles.

**Figure 11:**
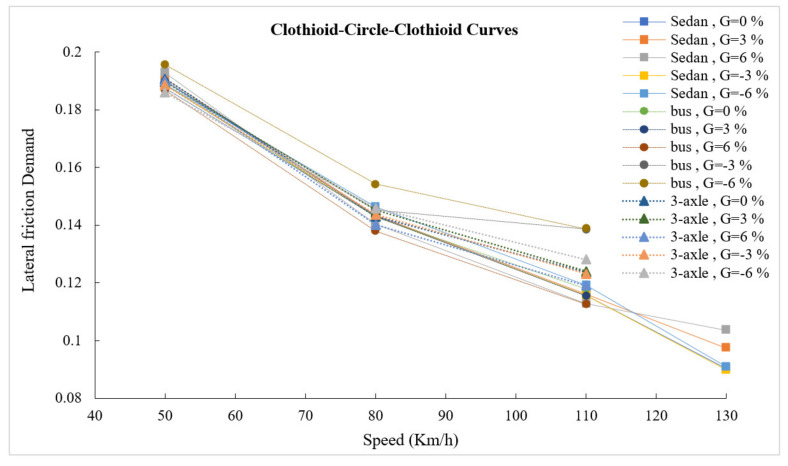
Lateral friction demand in clothoid–circle–clothoid curves.

Figure 11 shows the lateral friction demand for the designed clothoid–circle–clothoid curves. Except for the bus, in grades −3% and −6%, there is no significant difference between the sedan and heavy vehicles in values of lateral friction demand. The changes in the lateral friction demand, for all vehicles, on the clothoid–circle–clothoid curve are smaller than those on the simple circular curve. Consequently, the designed roads with a clothoid curve are recommended particularly in high-radius curves (curve with high design speed).

After comparing the simulation results of the clothoid–circle–clothoid and the simple circular curves, a maximum reduction of 13.9% was observed in the maximum lateral friction demand on a 951 m clothoid–circle–clothoid curve for the sedan. Furthermore, the lateral friction demand on 560, 252, and 79 m curves was reduced by 7.49%, 0.78%, and 0.8%, respectively. The maximum reduction in the lateral friction demand of the bus on 79, 252, and 560 m clothoid–circle–clothoid curves was 5.5%, 23.8%, and 14%, respectively. The corresponding values for a 3-axle truck were 4.1%, 17%, and 19.7%, respectively. Therefore, the effect of the clothoid transition curve on heavy vehicles is more evident than that on light vehicles.

### 3.3. Effect of Longitudinal Profile on Safety Margin

**Figure 12:**
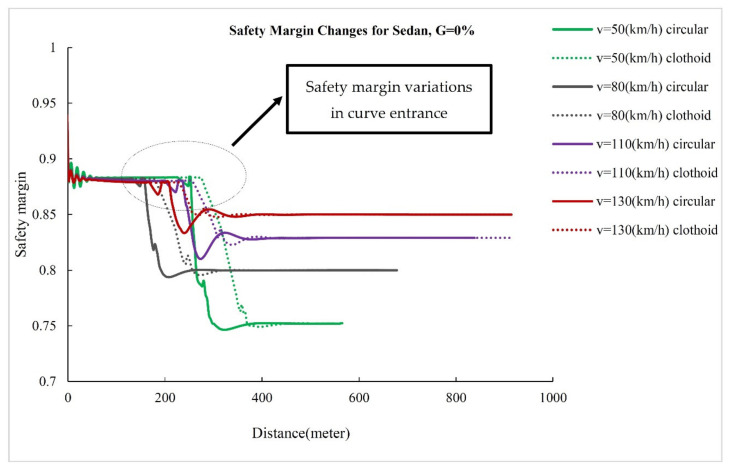
Safety margin rated for sedan.

**Figure 13:**
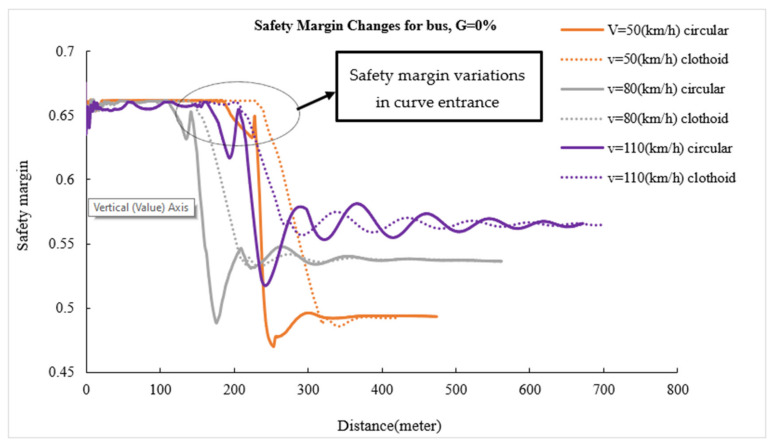
Safety margin rated for bus.

**Figure 14:**
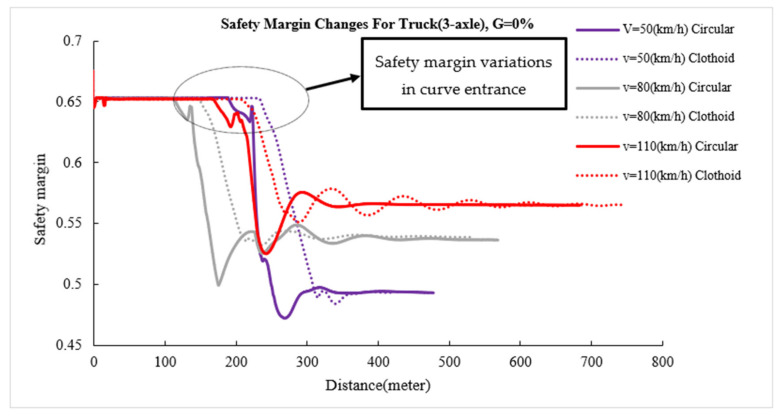
Safety margin rated for 3-axle truck.

The safety margin versus length diagram shows instantaneous changes in vehicle safety margin on various sections of horizontal curves. Figure 12, Figure 13, Figure 14 show the changes in the safety margins of designed vehicles on a zero longitudinal grade. Due to the similarity of the trends in different grades, the changes in the safety margin for simulated vehicles are presented at four speeds and only on a longitudinal grade of zero to compare the simple circular and clothoid–circle–clothoid curves. In fact, the slope of the safety margin changes for the designed vehicles on simple circular and clothoid–circle–clothoid curves shows the decreasing trend of safety margin at the entrance of the curves. As can be seen inFigure 12, Figure 13, Figure 14, the changes in the safety margin at the entrance of the clothoid curve are smaller than those in the simple circular curve (the black circle) inFigure 12, Figure 13, Figure 14. Moreover, the safety margin reduction on the clothoid curve takes place with a lower grade than that of the simple circular curve.

### 3.4. Effect of Radius on the Minimum Safety Margin

**Figure 15:**
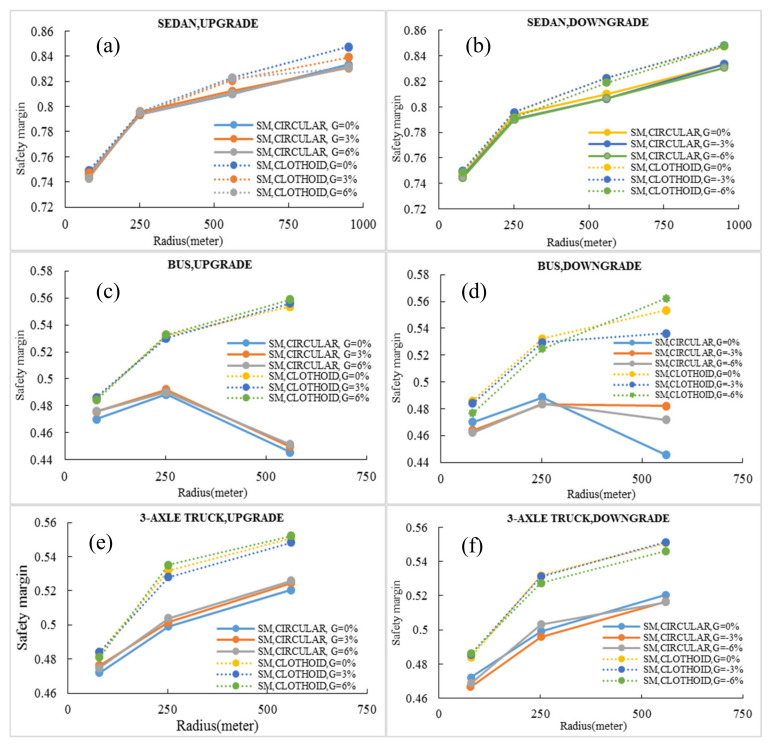
Minimum safety margin: (**a**) sedan in upgrades, (**b**) sedan in downgrades, (**c**) bus in upgrades (**d**) bus in downgrades, (**e**) 3-axle truck in upgrades, (**f**) 3-axle truck in downgrades.

Figure 15a,b shows the minimum safety margin of the sedan on upgrades and downgrades, respectively. As seen, the minimum safety margin increases with the increasing radius of the curve. The safety margin for 79 m simple circular and clothoid–circle–clothoid curves are approximately equal. By increasing the radius, the value of the safety margin in the simple circular curve differs from that of the clothoid–circle–clothoid curve. These values for the clothoid–circle–clothoid curve are more than those for the simple circular curve. Therefore, a clothoid transition curve should be designed with the increasing radius of the curve. For a sedan on a 951 m curve with a grade of 0%, the minimum safety margin on the clothoid curve increases by 1.65% compared to that of the simple circular curve.

The pattern of the minimum safety margin of the bus in the clothoid curves is similar to that of a sedan. However, the minimum safety margin of the bus first increases and then decreases in circular curves. There is a remarkable note in Figure 15c,d. If the value of the grade is −3%, the minimum safety margin of the bus will be constant after a specific radius. It means that the minimum safety margin probably will be independent after that specific radius. The finite conclusion about this subject will be obtained after simulating a wide range of radii. This issue is one of the future research works of authors. For the 3-axle truck, the pattern of the minimum safety margin variations is similar to the sedan’s diagram. There is only one difference. The minimum safety margin of the 3-axle truck in clothoid curves and for all ranges of radii is more than that of the circular curves. For the heavy vehicles (bus and 3-axle truck), the length of 951 m was not examined because in this length heavy vehicles slipped into the internal part of the curve.

Generally, by exact examination of Figure 15, the following results are evident:

- The difference between the minimum safety margin in circular and clothoid curves for heavy vehicles is greater than that of the sedan.
- The design of clothoid–circle–clothoid curves on the roads with a higher traffic volume of heavy vehicles leads to a greater safety margin for the vehicles.
- In low-radius curves, the difference in the safety margin of circular and clothoid is slight. Therefore, in lower radii, there is not a significant difference between circular and clothoid transition curves.

### 3.5. General Examination of the Safety Margin for Light and Heavy Vehicles

**Figure 16:**
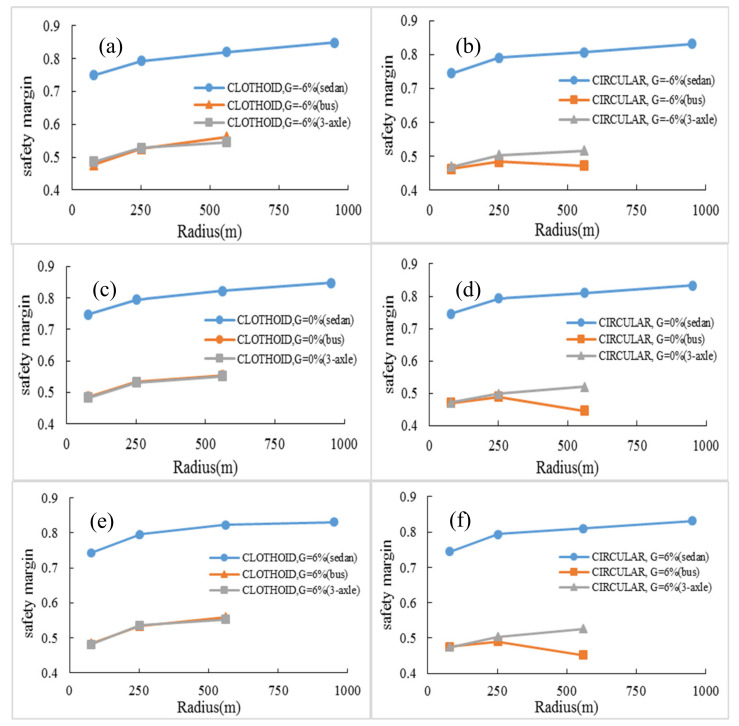
Comparison of safety margin for all three vehicles: (a) clothoid curve and G = –6%, (b) circular curve and G = –6%, (c) clothoid curve and G = 0%, (d) circular curve and G = 0%, (e) clothoid curve and G = 6%, (f) circular curve and G = 6%.

Preliminary differences of the safety margin parameter in the simple circular and clothoid–circle–clothoid curves, with various longitudinal upgrade and downgrade, were identified in the previous section. In this section, the safety margin for selected vehicles in this study is examined when driving these two curves. Figure 16 shows the changes in the safety margin against the radius for different longitudinal grades, in all three types of vehicles under study and for both horizontal curves.

Examination of Figure 16 reveals significant points, which are presented below:

- On all selected grades, the difference between the values of the safety margin for heavy vehicles (bus and 3-axle truck) is much more than that of the sedan (about 37%). This difference is related to the condition in which the road surface is dry (AASHTO assumption), but if the road surface conditions change, such as due to rainfall, this difference in value could change.
- For the bus, the pattern of changes in the values of the safety margin parameter, in different radius and on the clothoid–circle–clothoid curves, is almost constant and similar to that of the 3-axle truck vehicle (see Figure 16a,c,e). However, in circular curves (see Figure 16b,d,f), this value decreases with an increasing curve radius from 252 to 560 m (about 14%).
- In all conditions, the pattern of the safety margin for the 3-axle truck is constant. More radius leads to more safety margin.

## 4. Validation

This section of the paper examines the validation of the simulation’s results. Validation was conducted in two steps: (1) validation based on longitudinal forces and (2) validation based on lateral forces. Firstly, the authors selected references for validating their simulation. These references were the study conducted by Li et al. [31] for longitudinal forces, and also the study conducted by Li and He [18] for lateral forces.

**Figure 17:**
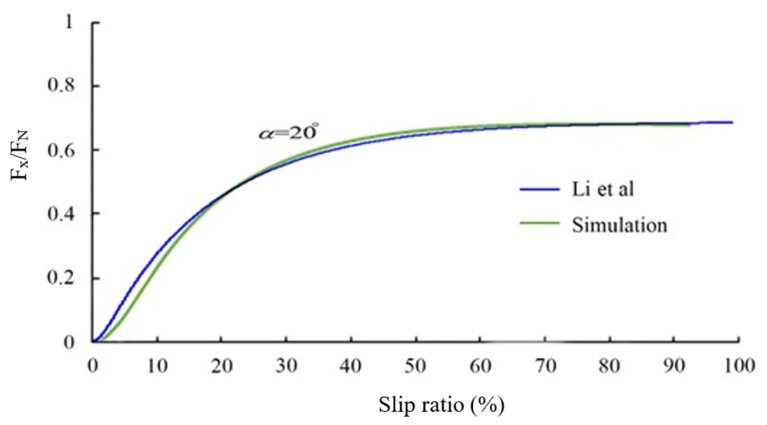
Comparing F_x_/F_N_ variations versus different slip ratio between the simulation values and experimental data.

**Figure 18:**
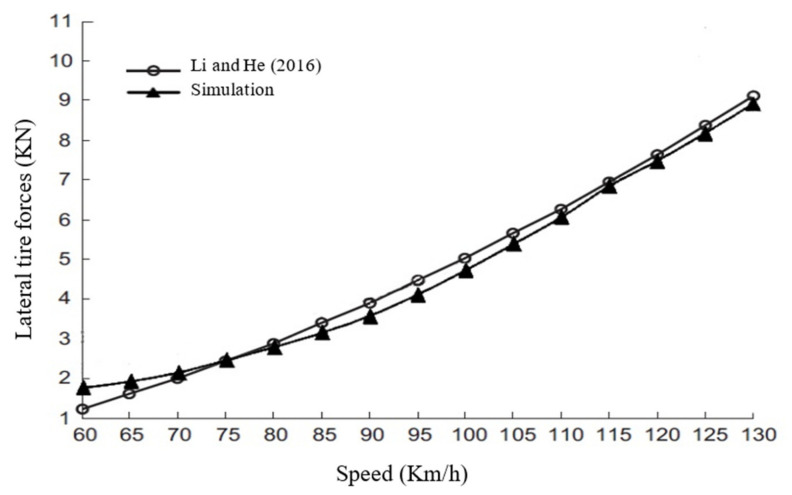
Comparing lateral forces versus speed between the simulation and experimental data.

The results of the Pacejka Model, simulated by the ADAMS/Car, were compared by experimental data of the study by Li et al. For experimental data, Li et al., plotted “Longitudinal Force/Normal Force versus Longitudinal Slip” diagrams for a sideslip of 20 degrees [31]. To validate the values of longitudinal forces, the authors cited Figure 2 of the study by Li et al. Similar to thementioned figure in the study of Li et al., the authors drew a diagram for their study. Both diagrams can be seen in Figure 17. This figure verified that the results obtained from simulation with ADAMS/Car in this study coincide with the results of Li et al. In the second step, the lateral force was used as the criteria of validation. For a constant radius and with different speeds, simulation of the tire was done. The lateral forces attained from the simulation were compared with the results of the study by Li and He in Figure 18. Similar to Figure 17, this figure also verified that the simulation conducted in this study is reliable.

## 5. Conclusions

Safety always is the most important aim that engineers and designers of roads follow. Most of the accidents on roads occur in horizontal curves. Therefore, the guarantee of safety in these parts of roads is essential. The principal model in the designing of horizontal curves (Equation (1)) has had some questionable points. This equation does not consider three important issues: the changes in weight and dimensions of vehicles, the coincidence of longitudinal grades with the horizontal curve, and differences in driver behavior. This study aimed to assess these issues. In fact, it can be said that the main concern of the authors was the improvement of safety in horizontal curves. On the other hand, one of the ways of increasing safety is the use of transition curves in geometric designs.

Consequently, for evaluating the proposed method of AASHTO standard, the lateral friction supply and lateral friction demand at various speeds of 50, 80, 110, and 130 km/h were calculated. Based on the obtained results, the margin of safety was determined for both simple circular and clothoid–circle–clothoid curves. Horizontal curves with different characteristics were simulated by ADAMS/Car. Moreover, various longitudinal grades (−6%, −3%, 0%, 3%, and 6%) were designed in the location of the horizontal curves. The basis of this simulation was the dynamic responses of vehicles. The results can be summarized as follows:

- The comparison of the values of safety margin in both curves indicates that the existence of the clothoid in curves with a low radius does not have a considerable impact on increasing safety margin. In contrast, in curves with a high radius, clothoid can improve the safety margin.
- For all vehicles and on the small radii, the safety margin values of both horizontal curves showed a small difference. However, by increasing the radius, the clothoid–circle–clothoid curve had better safety margin values than the simple circular curve, especially for heavy vehicles. As a result, it is a necessity to use a clothoid transition curve in the curves with a high radius. Albeit, in the case of high radii, the safety margin in both curves was nearly equal for the sedan.
- On simple circular curves, instantaneous changes in the safety margin are much greater than those in the clothoid–circle–clothoid curves. The reduction of the safety margin on the clothoid–circle–clothoid curves is much less than that on the simple circular curves.
- Most studies related to lateral friction factors have focused on simple circular curves, and there is no study related to the combination of horizontal and transition curves. In this study, the friction factors were applied to the clothoid–circle–clothoid system. These friction factors can be used as a control regulation of safety in geometric design for all combinations of curves. In fact, the proposed methodology can be applied for evaluating safety in any new form of curves that will be presented by engineers.
- The maximum lateral friction demand for simple circular curves indicate that the obtained friction demand for a sedan on low-radius curves does not significantly differ from those proposed by AASHTO. However, for heavy vehicles (bus and 3-axle truck), the maximum lateral friction factor showed more critical values for vehicle safety. Therefore, the designers must differentiate between light and heavy vehicles.
- According to the results, the maximum lateral friction factor decreases with the increasing radius in both horizontal curves.
- According to the maximum lateral friction factor for simulated longitudinal grades, a maximum reduction of 13.9% was observed in the maximum lateral friction for a sedan on a 951 m clothoid curve. The corresponding reductions to 560, 252, and 79 m curves were 7.49%, 0.78%, and 0.8%, respectively. The maximum reduction of the lateral friction factor on a 79, 252, and 560 m clothoid curve for the bus was respectively 5.5%, 23.8%, and 14% relative to a simple circular curve. The corresponding values for a 3-axle truck on the designed curves were 4.1%, 17%, and 19.7%, respectively. The comparison of these values indicates that the impact of the transition curve on heavy vehicles is more significant than that on light vehicles. As the final part of the paper, the authors have two attractive proposals for future studies. By more examination of the point-mass model, it can be found that the model has two other major challenging issues:
- In the point-mass model, vehicles are considered as a point mass. However, a vehicle has four tires (or more) that can have various behaviors. Therefore, a new proposal that authors have selected for their future studies is to analyze the behavior of various tires of design vehicles.
- The AASHTO green book assumes that superelevation is constant in simple circular curves. On the other hand, this standard proposes that a part of superelevation implementation distance occurs inside the curve. Therefore, the initial and final parts of the curves have different superelevation than the middle part. This issue can create safety challenges for vehicles, especially vehicles with long length. Therefore, another suggested proposal of the authors is related to superelevation in simple circular horizontal curves.

## Figures and Tables

**Table 1:** The design values used in the study.

Speed (km/h)	The Radius of the Circular Curve (m)	Length of Clothoid Curve (m)
50	79	71
80	252	91
110	560	114
130	951	134

**Table 2:** Geometric design specifications based on American Association of State Highway Transportation Officials (AASHTO).

Design Speed (km/h)	Superelevation (%)	Maximum Friction [12]	Total $\left(\frac{e}{100}+f\right)$	Calculated Radius (m)	Rounded Radius (m)
50	6	0.19	0.25	78.7	79
80	6	0.14	0.2	252.0	252
110	6	0.11	0.17	560.4	560
130	6	0.08	0.14	950.5	951

**Table 3:** Input parameters in the ADAMS/Car for simple circular and clothoid–circle–clothoid curves.

Parameters in Simple Circular Curves							
Speed (km/h)	Curve Radius (m)	Longitudinal Grade (%)	Superelevation (Degree)	Friction Coefficient of Road Surface	Road Width (m)		
50	79	6, 3, 0, −3, −6	3.42	0.9	3.65		
80	252	6, 3, 0, −3, −6	3.42	0.9	3.65		
110	560	6, 3, 0, −3, −6	3.42	0.9	3.65		
130	951	6, 3, 0, −3, −6	3.42	0.9	3.65		
Parameters in Clothoid–Circle–Clothoid Curves							
Speed (km/h)	Curve Radius (m)	Longitudinal Grade (%)	Superelevation (Degree)	Friction Coefficient of Road Surface	Road Width (m)	Length of Clothoid (m)	Length of Runoff (m)
50	79	6, 3, 0, −3, −6	3.42	0.9	3.65	38	33
80	252	6, 3, 0, −3, −6	3.42	0.9	3.65	48	43
110	560	6, 3, 0, −3, −6	3.42	0.9	3.65	61	53
130	951	6, 3, 0, −3, −6	3.42	0.9	3.65	72	62

**Table 4:** Vehicle characteristics for simulation.

Vehicle Type		Sedan	Bus	3-Axle Truck
Parameter	Length of wheel-base (mm)	3076	10,268	8047
	Width (mm)	1895	2483	2244
	Height (mm)	1500	3411	3132
	Weight (kg)	1980	11,697.1	10,844.3
	Gravity Center from Ground (mm)	450	1300	1163

**Table 5:** The main factors in tire–road interaction friction.

Parameters	Tire		
	Sedan	Bus	3-Axle Truck
Type	pac2002-235-40R18	pac2002-255-40R19	pac2002-255-40R19
Inflation pressure (MPa)	200,000	800,000	800,000
Rim diameter (in)	17	22.5	22.5
Width (m)	0.235	0.318	0.318
Unloaded radius (m)	0.326	0.548	0.548
Pattern design	MF *	MF *	MF *
F_N_ (N)	5000	30,000	30,000
Slip angle (Degree)	0 to 20	0 to 20	0 to 20
Longitudinal slip (%)	0 to 100	0 to 100	0 to 100
Road			
Microtexture	Drainage capability	Road composition	Lane grooves
Asphalt	Assumed good	Simple circular and clothoid–circle–clothoid curves	Flat and smooth

* Note: MF = Magic Formula, combine method, intermediary layers = 0.9 (dry friction).

**Table 6:** Values obtained for potential friction coefficient (f_i max_).

f_i max_	Sedan (pac2002-235-40R18)	Bus and 3-Axle Truck (pac2002-255-40R19)
f_x max_	1.051947	0.73117
f_y max_	0.93899	0.675256
